# Automatic measurements of volar tilt and radial inclination of the distal radius on 3D models: validation against manual methods

**DOI:** 10.1038/s41598-025-33737-6

**Published:** 2026-01-06

**Authors:** Emilia Gryska, Katleen Libberecht, Johan Andersson, Charlotte Stor Swinkels, Peter Axelsson, Anders Björkman

**Affiliations:** 1 Department of Hand Surgery, Sahlgrenska University Hospital, Länsmansgatan 20, Mölndal, 431 30 Sweden; 2 Institute of Clinical Sciences, Sahlgrenska Academy, University of Gothenburg, R-huset, plan 7, Mölndals sjukhus, Göteborgsvägen 31, Mölndal, 431 80 Sweden; 3 Department of Medical Physics and Biomedical Engineering, Sahlgrenska University Hospital, Blå stråket 9A, Gothenburg, 413 46 Sweden

**Keywords:** Distal radius, 3D, Automatic, Volar tilt, Radial inclination, Anatomic landmarks, Anatomy, Engineering, Health care, Medical research

## Abstract

Assessing distal radius malunions traditionally relies on measuring volar tilt and radial inclination in 2D radiographs. Reliability of these parameters is limited by projection errors and landmark ambiguity. Using 3D models allows more precise quantification of these parameters. This study validated an algorithm for measuring volar tilt and radial inclination on 3D virtual models of malunited and healthy radii. The algorithm establishes a 3D coordinate system and anatomical landmarks replicating conventional 2D definitions to maintain clinical compatibility. Automatic 3D measurements on models from 16 participants were compared with manual measurements on 2D radiographs (by three surgeons in consensus) and on 3D models (by two independent raters). Agreement was assessed using Bland–Altman analysis, and landmark discrepancies were quantified. Mean differences between automatic 3D and manual 2D measurements were small: for volar tilt, 2.0° in malunited and 3.5° in healthy radii; for radial inclination, 1.5° and 2.3°, respectively. Strong agreement was also found between automatic and manual 3D measurements. Landmark deviations ranged from 0.5 to 2.8 mm, with an average longitudinal axis difference of 2.5°. This open-source algorithm provides a reproducible 3D assessment of distal radius alignment. By closely matching 2D standards, the algorithm has a high potential for clinical integration.

**Supplementary Information:**

The online version contains supplementary material available at 10.1038/s41598-025-33737-6.

## Introduction

 Three-dimensional (3D) virtual surgical planning (VSP) and the use of patient-specific surgical guides have been increasingly adopted, particularly for reconstructing complex malunions in the upper extremity^1–4^. Compared with conventional two-dimensional (2D) planning and freehand osteotomy, 3D planning has demonstrated superior accuracy of correction^1,5^ and more favourable functional outcomes^2^. This is likely because planning corrections on 3D models facilitates more anatomically precise reconstructions than when relying on 2D images and radiographic parameters from these images for planning.

Although 2D radiographic measurements are considered the gold standard in preoperative planning, they suffer from low reliability^6–9^. Studies by Fox et al. and MacDermid et al. have demonstrated that providing detailed measurement instructions can improve consistency^7,10^, however, measurement protocols are not well standardized^6,10^. Furthermore, image quality issues, such as inaccurate projections^11–13^, cast artefacts, healed fracture lines, or obscured anatomical landmarks, may compromise measurement accuracy, especially in complex skeletal deformity^7^. Due to these limitations, 2D methods may not reliably measure the true extent of a malunion or the correction outcome, especially within clinically acceptable ranges of 5° for volar tilt and 2 mm for ulnar variance^14–16^. 3D measurement techniques offer a promising alternative, having shown good-to-excellent inter- and intra-rater reliability^17–19^.

While 3D measurements are generally more reproducible than 2D, manual placement of landmarks to estimate 3D radiographic parameters is affected by inter-rater variability, especially for volar tilt^20^. Algorithms that automatically identify landmarks and calculate radiographic parameters offer a promising solution to reduce this variability^19,21–24^. A few studies have demonstrated the reliability of automatic 3D methods compared with manual approaches in healthy radii^21–23^ and one in both malunited and healthy radii^19^.

For automatic 3D measurement methods to be clinically relevant and meaningful, the anatomical landmarks and definitions used to compute radiographic parameters must correspond to those used in standard 2D assessments. This will ensure that 3D measurements remain directly comparable to established clinical guidelines and familiar to clinicians. A suitable method must also be validated on both healthy and malunited radii to demonstrate robustness in the full spectrum of clinical presentations. However, existing literature describing automatic algorithms for distal radius assessment does not fulfil these requirements. Previous studies show substantial variability in how anatomical landmarks and coordinate systems (CS) are defined for 3D measurements. Most approaches^17,22,23^ adopt the CS for the entire radius as recommended by the International Society of Biomechanics (ISB)^25^. Since the ISB CS was developed for rotation analysis, it differs from the conventional 2D reference system used for measuring distal radius malunion. Although Suojärvi et al.^24^ proposed an automated algorithm that replicated 2D measurement geometry on 3D models, it was validated only on healthy radii. Winter et al.^19^, on the other hand, evaluated an automatic 3D method on both healthy and malunited radii; however, their CS and measurement definitions diverged notably from standard 2D radiographic practice^7^. Consequently, as current algorithms diverge from clinical measurement definitions or lack validation in malunited cases, they cannot be regarded as clinically applicable for improving the reliability of radiographic assessment in distal radius fractures or malunions.

This study aimed to develop an algorithm replicating conventional 2D definitions to maintain clinical compatibility for the automatic measurement of volar tilt and radial inclination on 3D virtual models of both malunited and healthy radii. The algorithm was validated against standard 2D measurements obtained from conventional radiographs, as well as manual 3D measurements on virtual models. This validation assessed whether the anatomical landmarks previously proposed to best approximate 2D measurements in healthy radii^24^ maintain the same relationship in both malunited and healthy radii.

## Methods

### Patient data

This study utilized virtual radius models generated for 3D surgical planning of sixteen participants who underwent treatment for symptomatic extra-articular distal radius malunion at Sahlgrenska University Hospital between July 2021 and July 2023. The radius models were generated in Mimics (*Materialise*, NV Leuven, Belgium) from segmentation masks created semiautomatically by a hand surgeon (KL). The segmentation was performed using a predefined bone threshold, the Smart Fill function, and manual adjustments in CT images acquired by a GE Discovery CT750 HD scanner. The image slice thickness was 0.625 mm, and the pixel size was 0.39 mm × 0.39 mm^26^. Conventional preoperative radiographs of the malunited and healthy arms were obtained from the hospital’s radiology image archiving system for all participants, except one, for whom images were not available. Additionally, two participants had only radiographs of the malunited arm available. The use of the data was approved by the Swedish Ethical Review Authority (DNR-2021-01974), and the study was performed in accordance with the Declaration of Helsinki. All patients gave written informed consent after receiving oral and written information about the study.

### Data collection

#### Manual 2D measurements

**Fig. 1:**
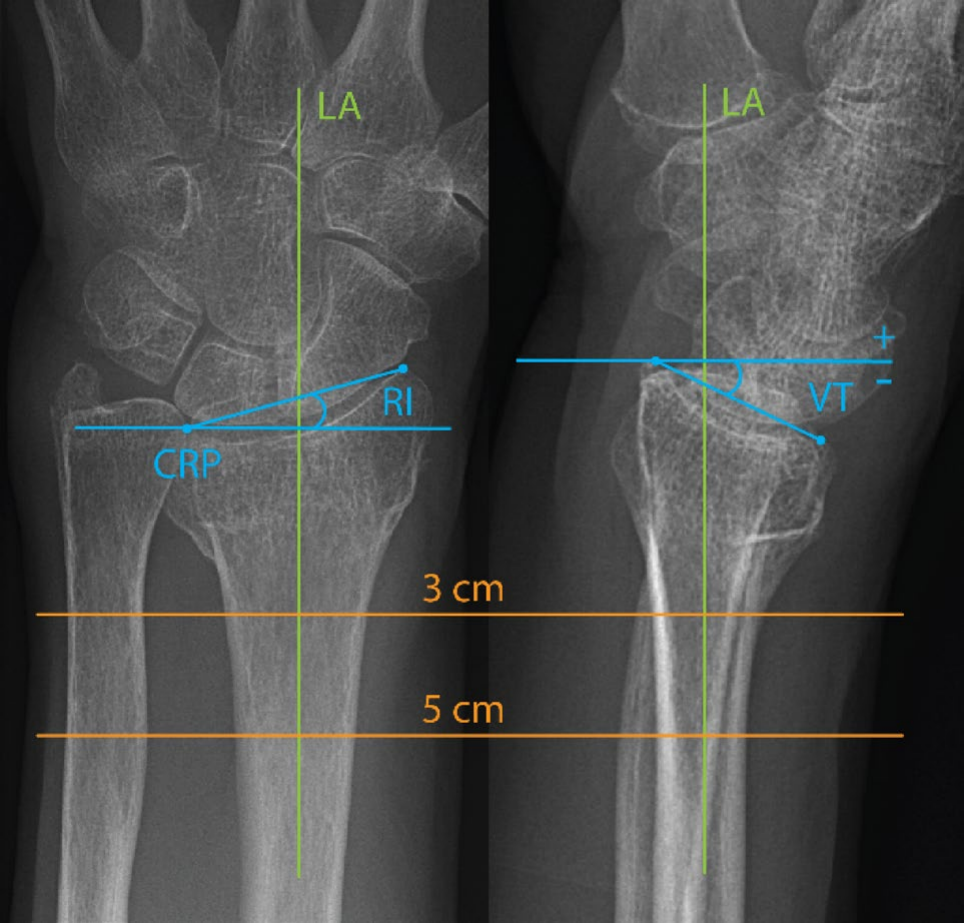
Measurement of radial inclination (RI) and volar tilt (VT) on standard radiographs. The longitudinal axis of the radius (LA) is defined as the central line through the radial shaft between 3 and 5 cm proximal to the central reference point (CRP). The CRP is located midway between the volar and dorsal ulnar corners of the articular surface. Radial inclination is measured on the postero-anterior view as the angle between the line drawn from the CRP to the radial styloid and a line perpendicular to the LA. Volar tilt is measured on the lateral view as the angle between the line connecting the volar and dorsal rims of the articular surface and a line perpendicular to the LA. A negative volar tilt value indicates a dorsal angulation.

Three expert^27^ hand surgeons (KL, AB, and PA) measured volar tilt and radial inclination in the available conventional radiographs using Agfa Enterprise Imaging digital measurement tools. The final volar tilt and radial inclination values were established during a meeting attended by all three surgeons. First, the surgeons reviewed the measurements according to Kreder et al.^9^, followed by discussing each case until a consensus was reached. The volar tilt was measured in standard lateral radiographs as the angle between a line perpendicular to the central long axis of the radius and a line connecting the volar and dorsal edges of the articular surface of the distal radius^9^ (Fig. 1). The radial inclination was measured in standard posterior-anterior radiographs as the angle between a line perpendicular to the central longitudinal radius axis and a line connecting the radial styloid and a point midway between the volar and dorsal ulnar corners of the distal radius, known as the central reference point (CRP)^28^ (Fig. 1).

#### Manual 3D measurements

**Fig. 2:**
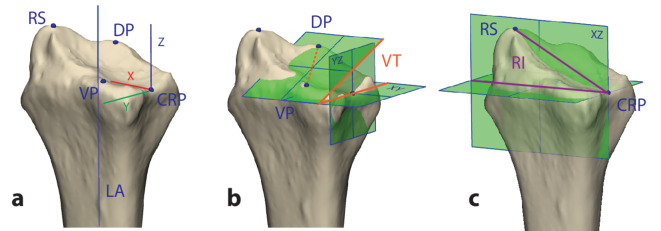
Three-dimensional anatomical coordinate system and landmarks used for manual measurement of volar tilt (VT) and radial inclination (RI). (**a**) Anatomical landmarks include the central reference point (CRP), radial styloid (RS), most volar point on the articular surface (VP), and the most dorsal point on the articular surface (DP); (**b**) Volar tilt is defined as the angle between the XY-plane and the projection of the line connecting the VP and DP (dashed orange line) on the YZ-plane (full orange line). (**c**): Radial inclination is defined as the angle between the line from the RS to the CRP and the XY-plane.

Two raters: an engineer experienced in 3D VSP (CSS – R1) and one of the hand surgeons (KL – R2), manually measured the volar tilt and radial inclination on the malunited 3D virtual radius models by identifying relevant landmarks on the 3D virtual radius models (Fig. 2a). R1 additionally measured the volar tilt and radial inclination on the healthy 3D virtual radius models. KL also participated in 2D measurements, conducted at least a month after the 3D measurements. Therefore, although not blinded, the risk of recognition bias was minimal. The volar tilt was defined as the angle between two specific planes: the first, called the XY-plane, was perpendicular to the central longitudinal axis of the radius and passes through the CRP (Fig. 2b)^28^; the second plane passed through the most dorsal point of the articular surface and the most volar point of the distal radius, also known as the “teardrop” region on radiographs^29^ and was perpendicular to the YZ-plane. The longitudinal axis was defined as the central line in the distal radius segment between 3 and 5 cm measured proximally from the CRP in both the frontal and lateral projections of the 3D model. The radial inclination was measured as the angle between the XY-plane and a line connecting the radial styloid to the CRP (Fig. 2c).

#### Automatic 3D measurements

Volar tilt and radial inclination were also measured automatically on both malunited and healthy 3D virtual models of the radius. The algorithm proposed here and published online (https://github.com/emiliagyska/X3mityRCS, commit 0c1c435) is knowledge-based^22^, meaning that it relies on identifying landmarks by analysing the virtual bone model’s geometry. The algorithm was developed through an iterative process to ensure accurate identification of landmarks in both severely malunited and healthy radii. We used landmarks recommended by Suojärvi et al.^24^, who found that the optimal Z-axis of a radius model aligned with the longitudinal axis of a segment located between 28.8 and 53.3 mm from the CRP. The landmarks on the most dorsal and volar cortical margins – located at 33% and 57% of the normalized margin length from the CRP, respectively – yielded 3D volar tilt measurements that most closely matched 2D volar tilt values^24^. Dorsal and volar cortical margins refer to the outermost cortical bone edges on the dorsal (posterior) and volar (anterior) aspects of the distal radial metaphysis and articular surface. These margins form the dorsal and volar “rims” of the distal radius that are visible on lateral radiographs and are routinely used to define the line of the articular surface when measuring volar tilt. Their identification is clinically important because the measured volar tilt depends directly on the relative positions of these two cortical edges; inaccurate localisation can lead to substantial variation in the angular measurement. In 3D models, replicating these same cortical margins ensures that automatically computed volar tilt corresponds to established 2D radiographic definitions and remains clinically interpretable.

The landmarks recommended by Suojärvi et al.^24^ were established for healthy wrists only, but their method was not directly compared with conventional 2D measurements, only references from previous studies. Therefore, we validated our algorithm on both healthy and malunited radii. In the following, we describe the general principles used in the algorithm to identify the landmarks and to define the anatomical CS in 3D.

**Fig. 3:**
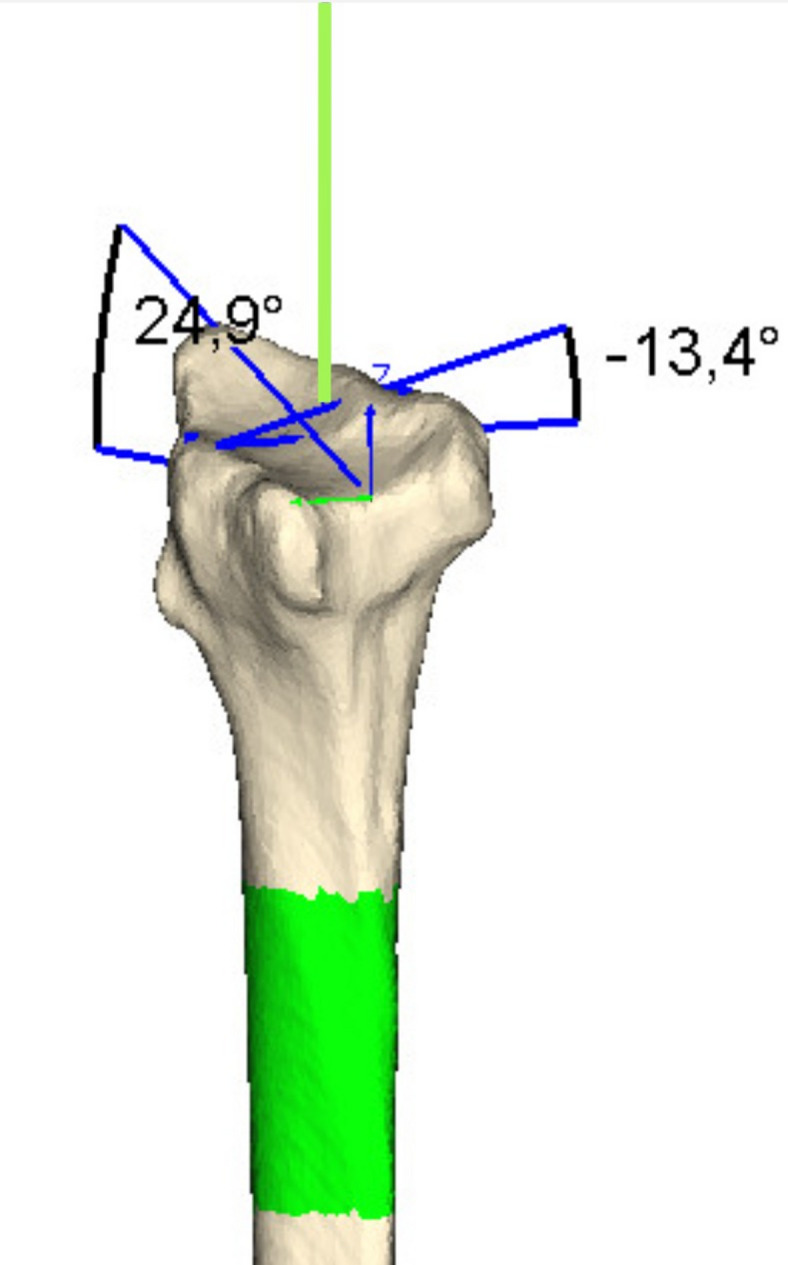
A screenshot from the software showing a virtual radius model with reference lines used to calculate volar tilt (−13.4°) and radial inclination (24.9°). The green line extending distally from the model indicates the longitudinal axis of the radial shaft located between 28.8 and 53.3 mm from the CRP. A negative volar tilt value indicates a dorsally tilted malunion.

The algorithm identifies the anatomical landmarks required to compute volar tilt and radial inclination through a sequence of knowledge-based steps (see the supporting images in the Supplement). When an STL model of the entire radius is imported, it initially appears in an arbitrary orientation within the 3D CS. To standardise its orientation, the algorithm determines the proximal–distal, radial–ulnar, and dorsal–volar directions as follows:

1. Alignment of the longitudinal axis: the algorithm estimates the model’s longitudinal axis and computes the centre of mass along this axis. The model is then rotated to align its longitudinal axis with the Z-axis of the CS and translated such that the centre of mass coincides with the origin.
2. Identification of proximal and distal ends: two cross-sectional curves, perpendicular to the Z-axis, are generated at 8% and 92% of the model’s total length. The curve with a more circular geometry is classified as the proximal end, while the other corresponds to the distal end of the radius. If the proximal end is oriented toward the positive Z-direction, the model is rotated about the origin so that the proximal end aligns with the negative Z-direction.
  For clarity, in this study, the “ulnar direction” refers to the mediolateral direction pointing toward the ulna, and the “radial direction” refers to the direction toward the radial styloid. Together, these define the radial–ulnar axis of the distal radius. In the anatomical coordinate system constructed by the algorithm, this axis corresponds to the X-axis, with the positive X-direction oriented radially and the negative X-direction oriented ulnarly.
3. Determination of the radial–ulnar and dorsal–volar axes: a third cross-sectional curve is generated at 35% of the model’s length measured from the proximal end. The most prominent inflection point on this curve is used to identify the ulnar direction. A line perpendicular to the Z-axis and passing through this point defines the radial–ulnar axis. The model is then rotated about the origin so that the X-axis is parallel with this line (negative X directed ulnarly), and the Y-axis is oriented positively in the dorsal direction.
  After this procedure, the model is standardised within the CS such that the origin corresponds to the centre of mass, the positive X-axis points radially, the positive Y-axis points dorsally, and the positive Z-axis points distally. Next, the algorithm identifies the radial styloid through the following steps:
4. Finding the distal articular surface: The model is first translated along the Z-axis so that the CS origin coincides with the distal cross-sectional curve identified in step 2 at 8% of the model’s length from the distal end. The axis of inertia is then calculated for this distal part of the model, which currently includes all triangles with a positive Z-coordinate. This inertia axis approximates the direction of the articular surface. The entire radius model is translated again along the Z-axis to place the origin on the articular surface, and then rotated about the origin so that the inertia axis aligns with the X-axis.
5. Initial positioning of the distal articular surface: the model is translated along the Z-axis so that the origin coincides with the distal cross-sectional curve identified previously (8% from the distal end). An inertia axis is then computed for the distal portion of the model, defined as all triangles with positive Z-coordinates. This axis approximates the orientation of the articular surface. The radius is translated again to position the origin directly on the articular surface and subsequently rotated about the origin to align the inertia axis with the X-axis.
6. Reorientation using a distal cross-section: a cross-sectional curve is generated in the YZ-plane, and its centre of mass and inertia axis are calculated. The model is translated to place the origin at this centre of mass and rotated so that the inertia axis aligns with the Y-axis.
7. Refining the distal portion: the distal portion is redefined to include only triangles with Z-coordinates greater than − 1 mm.
8. Iterative refinement: steps 6 and 7 (above) are repeated five times. After iteration, the model is translated along the Z-axis so that the origin lies just distal to the articular surface.
9. Cross-sectional refinement in the XZ-plane: triangles with Z-coordinates greater than − 5 mm are selected, and a cross-section of these triangles is created in the XZ-plane. The centre of mass and the inertia axis of this section are calculated. The model is translated so that the origin coincides with the centre of mass and rotated to align the inertia axis with the X-axis.
10. Final iteration and styloid detection: step 9 is repeated five times. The radial styloid is then identified as the point with the maximum Z-coordinate among all points located on the radial (positive X) side.
  The ulnar volar and dorsal corners of the distal radial articular surface, as well as the CRP, are then identified as follows:
11. Locating the ulnar dorsal and volar corners: the model is rotated 25° about both the X- and Y-axes. The point with the highest Z-coordinate in the ulnar (negative X) direction is identified as the ulnar dorsal corner. The model is returned to its orientation after step 9, then rotated by −25° about the X-axis and 25° about the Y-axis. The point with the highest Z-coordinate in the ulnar direction is identified as the ulnar volar corner. The model is returned to its orientation after step 10 and subsequently translated along the X- and Y-axes so that the origin coincides with the midpoint between these two landmarks.
12. Identification of the CRP: the model is rotated 25° about the Y-axis, and triangles with Z-coordinates greater than − 10 mm are selected. A cross-section in the XZ-plane is generated from these triangles, and the point with the highest Z-coordinate is defined as the CRP. This point becomes the final origin of the coordinate system used to calculate radiographic parameters. The model is then returned to its post–step 10 orientation.
  To identify the most dorsal and volar cortical margins of the distal radius and the landmarks for calculating volar tilt, the algorithm executes the following steps:
13. Extraction of dorsal and volar margin curves: triangles with Z-coordinates greater than − 5 mm are selected. Twenty section curves are generated by intersecting these triangles with planes parallel to the ZY-plane, uniformly spaced between the origin and the styloid. To identify the dorsal margin, the model is rotated 5° about the X-axis, and the point with the highest Z-coordinate on each section curve is selected. A curve connecting all the lines indicates the dorsal margin. The model is returned to its baseline orientation and rotated − 5 ° about the X-axis to identify the corresponding volar margin points and curve. The model is then restored to its baseline orientation again.
14. Reference landmarks for volar tilt: a single reference point is selected on each margin curve: the volar point located at 33% of the normalised curve length from the CRP (where the distance along the margin between the CRP and the styloid is 100%), and the dorsal point located at 57% of normalised length from the CRP. These points define the measurement line for volar tilt.
  In the last stage, final adjustments are made to define the anatomical CS for the imported radius model:
15. Alignment using the radial shaft: the model is translated so that the origin coincides with the radial styloid. An inertia axis is computed for triangles located between − 60 mm and − 40 mm from the styloid, and the model is rotated about the origin to align the inertia axis with the Z-axis.
16. Final Z-axis alignment at the CRP: the model is translated so that the origin coincides with the CRP. An inertia axis is calculated for triangles located between − 53.3 mm and − 28.8 mm from the CRP, and the model is rotated so that this axis aligns with the Z-axis.
17. Refinement of the styloid and X-axis definition: the styloid position is refined by selecting the point with the highest Z-coordinate within a 2-mm radius of the previously identified styloid point. The X-axis is then defined as a line originating at the CRP, running parallel to a line passing through the styloid and the Z-axis, and perpendicular to the Z-axis.
  At this stage, the anatomical coordinate system is fully defined, with the origin at the CRP, the Z-axis aligned with the inertia axis of the mid-shaft region, the X-axis perpendicular to the Z-axis and directed toward the projected styloid, and the Y-axis orthogonal to both. Volar tilt is measured as the angle between a line connecting the reference points on the dorsal and volar margins and the Y-axis projected onto the YZ-plane (Fig. 3). Radial inclination is calculated as the angle between the X-axis and the line from the origin to the radial styloid (Fig. 3).

### Statistical analysis

Measurements in 2D and 3D were reported for each participant. Summary statistics were used to compare the results of volar tilt and radial inclination measurements using the various approaches. Measurements that were not available were excluded from the analysis. We further evaluated the agreement between the automatic 3D measurements, the standard 2D measurements, and the 3D manual measurements, separately for malunited and healthy radii using Bland-Altman plots^30^. In the agreement analysis, the 3D manual measurement for malunited radii was calculated as the mean of measurements by R1 and R2. Normality of the data was assessed using D’Agostino and Pearson’s tests^31,32^ implemented in *normaltest()* (*scipy*, version 1.13.1). Although the differences in the agreement analysis were not always normally distributed, we decided to use the standard Bland-Altman approach. We justified this choice with Bland and Altman’s reasoning that the effect of the distribution on the limits of agreement (LoA) is likely negligible when no relationship between the means and the difference is observed^33^. The uncertainty of the LoA was estimated according to the formula for the confidence interval for the 95% LoA: +/- 1.96 root(3/*n*)*SD, where *n* is the sample size, and SD is the standard deviation^30,34^.

The consistency and reproducibility of the automatic 3D measurements were evaluated using a test–retest procedure, in which the algorithm was applied twice to each malunited radius model and the resulting volar tilt and radial inclination values were compared between runs.

Outliers were defined as differences between automatically and conventionally (2D) measured values exceeding the clinically acceptable difference. The clinically relevant difference in volar tilt has previously been established at 5°, based on its correlation with function^14^. There is no specific value for radial inclination because a correlation between loss of radial inclination and function has not been established. However, we consider a difference of 5° relevant, as this reflects a reasonable accuracy requirement. The outliers were compared with manually measured values on 3D models to determine whether the values were more consistent across 3D measurements or manual measurements. In the case of the former, we supposed that the outlier was likely due to 2D measurement error. Conversely, in the latter case, the outlier was likely due to automatic measurement error. We further qualitatively inspected each measurement to identify potential sources of the outliers.

Finally, we analysed the discrepancy in the placement of the following landmarks: CRP, radial styloid, and the tips of the most dorsal and volar cortical margins. The discrepancy was measured as the Euclidean distance between corresponding points placed by each rater or the algorithm on the malunited radii. We also measured the angle difference between the longitudinal axes defined by each rater or the algorithm.

## Results

Sixteen participants (14 women) with a median age of 61 years (range: 21–77) were included. Thirteen had dorsally angulated radius malunions, and three had volarly angulated malunions.

In the malunited radii, 3D measurements yielded a lower mean volar tilt compared to 2D measurements, while in the contralateral healthy radii, 3D measurements showed higher mean volar tilt (Tables 1 and 2). Radial inclination was similar between 2D and 3D methods in the malunited radii, but it was consistently higher in 3D measurements in the healthy radii.

**Table 1:** Radiographic measurements of volar Tilt and radial inclination in the malunited radii. The last row shows the absolute mean (standard deviation) difference between the 2D and 3D measurements. A negative volar Tilt value indicates a dorsally Tilted malunion. N/A – not available, ∆2D – mean difference from the 2D measurement, R1 – rater 1, R2 – rater 2, AUTO – automatic measurements by the algorithm.

VT [°]					RI [°]			
ID	2D	3D AUTO	3D R1	3D R2	2D	3D AUTO	3D R1	3D R2
0	−27.2	−30.0	−33.3	−30.5	13.9	15.2	14.6	16.0
1	−18.4	−13.0	−11.8	−11.2	14.6	15.0	18.1	16.9
2	−5.7	−6.6	−4.7	−4.5	9.0	10.0	10.8	10.0
3	−23.4	−14.4	−21.0	−25.0	5.5	7.2	5.7	5.7
4	23.0	27.3	26.6	30.4	31.6	32.9	33.4	33.7
5	−9.6	−13.4	−13.3	−11.4	22.2	24.9	23.8	25.4
6	−25.7	−23.8	−22.1	−24.5	19.6	22.3	21.8	20.6
7	−33.7	−38.2	−30.3	−34.8	12.4	10.3	14.9	9.6
8	−16.0	−10.3	−13.0	−11.8	20.9	21.7	22.4	21.6
9	−15.4	−11.5	−8.2	−6.6	18.0	16.5	17.1	16.6
10	−23.6	−25.1	−25.5	−27.5	14.7	17.2	19.9	16.2
11	N/A	5.3	5.2	7.6	N/A	9.7	11.0	10.3
12	−16.4	−13.4	−14.0	−10.2	25.4	28.0	25.4	25.6
13	−23.6	−18.6	−19.9	−15.3	16.0	21.1	18.8	18.8
14	−19.5	−14.8	−12.7	−10.9	26.8	23.5	22.2	24.8
15	3.3	4.6	1.0	0.3	5.0	11.7	11.6	10.2
Mean (SD):	−15.5 (14.0)	−12.2 (15.3)	−12.3 (14.7)	−11.6 (16.0)	17.0 (7.6)	17.9 (7.3)	18.2 (6.8)	17.6 (7.5)
∆2D	–	2.0 (3.8)	2.0 (3.9)	2.6 (4.9)	–	1.5 (2.6)	1.6 (2.6)	1.1 (2.1)

**Table 2:** Radiographic measurements of volar Tilt and radial inclination in the healthy radii. A negative volar Tilt value indicates a dorsal angulation. The last row shows the absolute mean (standard deviation) difference between the 2D and 3D measurements. N/A – not available, ∆2D – mean difference from the 2D measurement, AUTO – automatic measurements by the algorithm.

VT [°]						RI [°]		
ID	2D		3D AUTO		3D R1	2D	3D AUTO	3D R1
0	10.4		12.5		8.5	22.6	24.2	23.9
1	5.5		15.6		14.0	21.9	26.1	26.8
2	−1.7		0.5		0.8	15.2	16.9	16.4
3	19.1		11.2		9.7	20.9	22.7	23.4
4	8.4		10.6		12.1	16.5	23.0	21.7
5	N/A		18.2		15.9	N/A	28.8	30.3
6	12.3		14.0		14.1	21.4	24.0	26.8
7	5.0		9.6		13.4	21.8	22.8	21.4
8	14.6		15.6		14.8	23.9	24.5	26.2
9	11.3		14.2		13.5	21.6	20.0	22.0
10	15.2		17.1		17.2	18.9	24.6	24.1
11	N/A		11.9		11.1	N/A	23.7	22.6
12	9.5		14.2		12.5	22.0	24.3	23.1
13	0.1		19.3		12.4	25.1	28.6	27.8
14	N/A		16.0		15.8	N/A	28.2	25.5
15	14.0		14.6		14.6	23.8	23.2	25.3
Mean (SD):	9.5 (6.0)		13.4 (4.4)		12.5 (3.9)	21.2 (2.8)	24.1 (3.0)	24.2 (3.2)
∆2D	–		3.5 (5.9)		2.6 (5.3)	–	2.3 (2.3)	2.6 (2.0)

Six outliers were identified in volar tilt measurements: three in the malunited group and three in the healthy group. One outlier in the malunited group (ID3) had a very proximal malunion, crossing the segment 3 to 5 cm proximal to the CRP. Therefore, the algorithm determined the Z-axis differently from the manual raters. The other outliers were attributed to inaccuracies in the 2D measurements due to the suboptimal quality of the radiographies. For radial inclination, two outliers were observed in the malunited group and two in the healthy group. All of these were minimal (within 7°) and regarded as errors in 2D measurements.

**Fig. 4:**
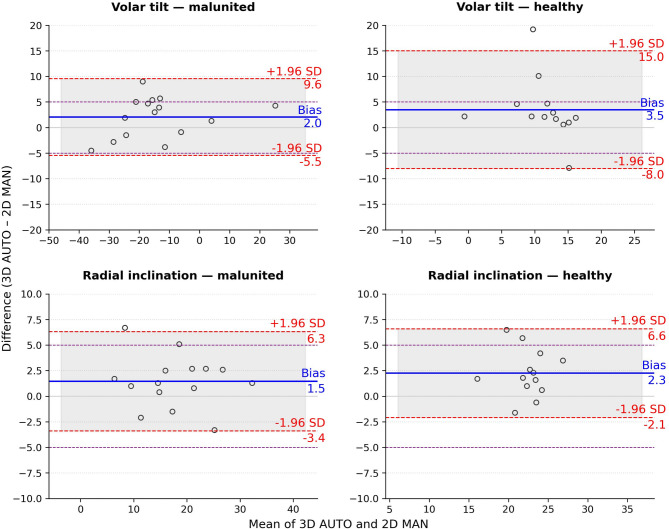
Bland-Altman agreement plot for volar tilt and radial inclination measurements between 2D manual (MAN) and 3D automatic (AUTO) measurements for malunited and healthy radii. The clinically relevant threshold of 5° is marked with a purple dashed line.

The mean difference (bias) between volar tilt measured automatically on 3D models and manually on 2D radiographs was 2.0° (SD: 3.8) in the malunited radii, which was similar or smaller than the differences between volar tilt measured manually on 3D models and manually on 2D radiographs (2.0°, [SD: 3.9] for R1 and 2.6° [SD, 4.9] for R2, Table 1). In healthy radii, the bias between automatic 3D and 2D volar tilt was 3.5° (SD 5.9), with LoA ranging from − 8° to 15.0°. All differences in volar tilt for the malunited radii were within the LoA (–5.5° to 9.6°), indicating closer agreement between 2D and 3D methods in the malunited group (Fig. 4). For radial inclination, the bias was 1.5° (SD 2.6) in malunited radii and 2.3° (SD 2.3) in healthy radii, with comparatively narrow limits of agreement (LoA) not exceeding 6.6° (Fig. 4). Due to a small sample size of *n =* 15 for the malunited and *n* = 13 for the healthy radii, the uncertainty of the LoA was large, 0.88*SD and 0.94*SD, respectively. In the malunited group, the LoA uncertainty amounted to 3.3° for volar tilt and 2.3° for radial inclination. In the healthy group, the values were 5.5° for volar tilt and 2.2° for radial inclination. Since the algorithm produced identical results in repeated measurement sessions, demonstrating 100% reproducibility, we attribute the large LoA to the low reliability of the standard 2D measurements.

On average, the algorithm required 676 ms to find the CS and calculate the parameters, using a computer equipped with an 11th Gen Intel^®^ Core™ i7-11800 H @ 2.30 GHz processor and 32 GB of RAM. No failures were reported when the user indicated whether the model was a left- or right-radius model. Automatic side detection, however, failed in four models, leading to erroneous volar tilt values.

**Fig. 5:**
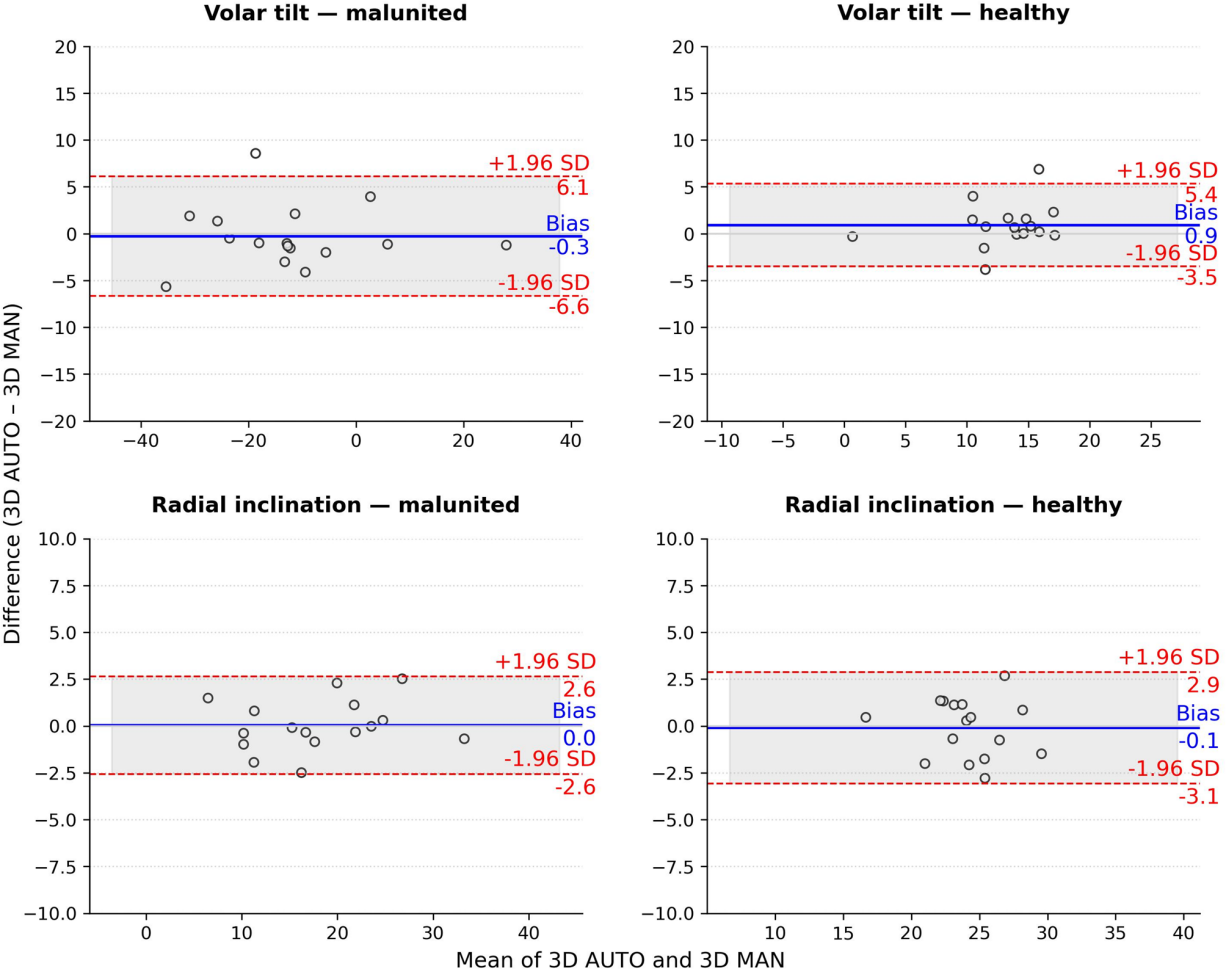
Bland-Altman agreement plot for volar tilt and radial inclination measurements between 3D manual (MAN) and 3D automatic (AUTO) measurements for malunited and healthy radii.

Bland–Altman analysis demonstrated a negligible mean bias in volar tilt between automatic and manual 3D measurements of −0.3° (SD 3.2) in malunited and 0.9° (SD 2.3) in healthy radii (Fig. 5), which was smaller than the bias observed between automatic 3D and 2D measurements. The bias in radial inclination for both malunited and healthy radii was also negligible (< 0.5°, SD:3.1, 1.3, and 1.5, respectively). The LoA were generally narrower between the two 3D methods than between the automatic 3D and 2D methods, indicating stronger consistency across 3D approaches. The LoA uncertainty was 0.85*SD, indicating the largest uncertainty for volar tilt of 2.7°.

The analysis of landmark placement on 3D models showed smaller discrepancies between the two raters than between each rater and the algorithm (Table 3). The largest mean Euclidean distance (2.8 mm) was observed for the dorsal edge landmark between R1 and the algorithm, with a maximum discrepancy of 9.6 mm. The greatest angular discrepancy in the Z-axis (2.5°) also occurred between R1 and the algorithm.

**Table 3:** Mean (standard deviation) and maximum discrepancy in landmark placement across all participants for the 3D measurements, as well as the difference in absolute volar Tilt and radial inclination values measured manually by raters (R1 and R2) and the algorithm (AUTO) on 3D malunited radius models. CRP – central reference point; Dorsal – the most dorsal point on the articular surface; Volar – the most volar point on the articular surface; Z axis – the longitudinal (Z) axis of the radius model; abs – absolute value; ∆RI – difference in radial inclination; ∆VT – difference in volar Tilt.

Comp. pair	CRP [mm]		Styloid [mm]	Dorsal [mm]	Volar [mm]	Z axis [°]	abs ∆RI [°]	abs ∆VT [°]
R1 v. AUTO		0.8 (0.6), max 1.9	1.3 (0.9), max 2.8	2.8 (2.7), max 9.6	2.4 (1.4), max 4.2	2.5 (1.8), max 6.3	1.5 (1.2), max 4.6	3.5 (3.7), max 13.9
R1 v. R2		0.7 (0.4), max 1.8	1.1 (0.8), max 2.9	1.8 (2.7), max 8.9	1.1 (0.9), max 2.9	1.8 (1.0), max 3.7	1.3 (1.4), max 5.3	2.6 (1.5), max 4.8
R2 v. AUTO		0.6 (0.3), max 1.4	1.2 (0.8), max 3.2	2.0 (1.3), max 5.1	2.4 (1.4), max 4.6	2.1 (1.5), max 6.3	1.1 (0.8), max 2.4	4.4 (3.8), max 15.8

**Fig. 6:**
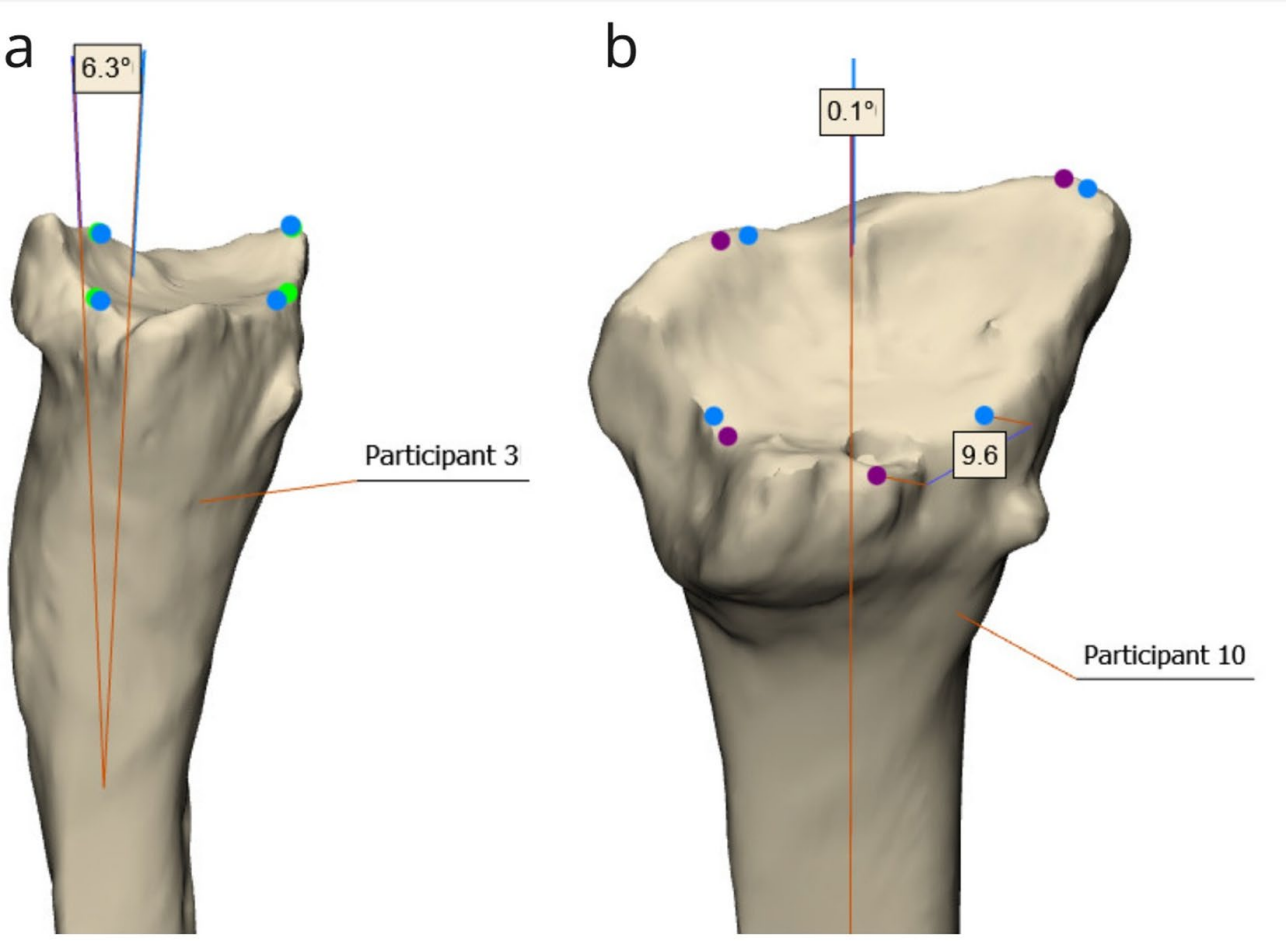
An example of a landmark placement discrepancy that led to the largest and the smallest differences in volar tilt measurements between the manual and automatic measurements. (**a**) Landmark discrepancy between R2 (green) and the algorithm (blue) that resulted in a 15.8° difference in volar tilt, and (**b**) between R1 (purple) and the algorithm (blue) that resulted in a volar tilt difference of 0.4°.

The largest discrepancy in volar tilt measurement of 10.6° between the algorithm and R2 occurred in Participant 3, which was identified as an outlier (Fig. 6a). Qualitative inspection revealed that the Z-axis discrepancy at 6.3° was the source of error due to malunion in the radius segment between 3 and 5 cm from the CRP. Conversely, a minimal volar tilt difference of 0.4° was found between R1 and the algorithm in Participant 10, despite a discrepancy of 9.6 mm in dorsal landmark placement. This discrepancy was almost entirely along the X-axis, which did not affect volar tilt calculation because volar tilt is derived from the angle between the Y-axis and the line connecting the dorsal and volar edges projected onto the YZ-plane (Fig. 6b).

## Discussion

Despite the increasing use of 3D imaging in distal radius assessment, the literature still lacks clinically aligned, sufficiently validated 3D measurement methods for volar tilt and radial inclination. Addressing this gap, the present study introduces and validates a fully automatic algorithm that reconstructs a 2D-equivalent anatomical coordinate system in both healthy and malunited 3D virtual radii and calculates volar tilt and radial inclination. In contrast to previous automatic methods, our approach is explicitly designed to mirror conventional 2D definitions and is tested in both normal and pathologic anatomy, thereby facilitating direct clinical interpretability and supporting translation of 3D measurements into everyday practice. The algorithm demonstrated 100% reproducibility on repeat testing. Agreement with 2D reference measurements was high, with mean differences in volar tilt of 2.0° for malunited radii and 3.5° for healthy radii, and in radial inclination of 1.5° and 2.3°, respectively. Landmark placement discrepancies between the algorithm and raters ranged from 0.5 to 2.8 mm, with a maximum angular deviation of 2.5° along the Z-axis.

The algorithm was based on distal radius landmarks described by Suojärvi et al.^24^ that replicate 2D measurements in healthy radius models. Interestingly, our algorithm showed a lower mean difference in volar tilt between the automatic 3D and 2D measurements (2.0°) in malunited radii but a larger difference of 3.5° in healthy radii. A smaller difference than in healthy radii between the 3D and 2D measurements (2.3°) was also noted for radial inclination in malunited radius models (1.5°). Since Suojärvi et al.^24^ compared their automatically acquired 3D values to literature reports rather than directly to 2D measurements, the actual difference between their 2D and 3D measurements is unknown.

Previous studies have reported systematic differences between 2D and 3D measurements, particularly in malunited radii^17,18,35^. Most of these studies found larger absolute values for 2D measurements compared to manual 3D measurements. Reported mean differences in volar tilt ranged from 2°^35^ to 3.7°^17^, while differences in radial inclination ranged from 1°^17^ to 5.7°^18^. Our findings, comparing manual 3D measurements with 2D measurements, are consistent with those reported in the literature. A direct comparison of the discrepancies between the 2D and 3D values across studies, however, is hindered by variability in the definition of the anatomical CS and the landmarks used to calculate 3D radiographic parameters. While we defined our 3D CS to resemble that used in 2D measurements, Miyake et al.^18^ and Smees et al.^17^ use the 3D CS proposed by the ISB^25^, which is defined primarily for motion analysis. Furthermore, the dorsal and volar ulnar tips of the rim of the sigmoid notch have been most commonly used to calculate volar tilt on 3D models^17,23,35^. In normal radii, using these landmarks likely underestimates the volar tilt values^24^ compared to more radial landmarks. This observation is supported by our mean 3D volar tilt values measured on the distal radius in healthy forearms (12.9° with the algorithm and 12.5° with R1), which are higher than those reported by Athlani et al. (8°–9°) using dorsal and volar ulnar tips of the sigmoid notch rim^35^. Since most results reported in the literature show larger absolute volar tilt values measured on 2D radiographs compared to those measured on 3D models, using sigmoid notch rim landmarks may underestimate volar tilt values not only in healthy but also in malunited 3D radii models. Another problem with the use of the volar and dorsal tips of the rim of the sigmoid notch is that they are rounded and not clearly defined on 3D models^36^. This poses a problem not only for the manual placement of the landmarks on 3D models but also for identifying these landmarks by a knowledge-based algorithm.

Despite a relatively small bias between the 3D and 2D measurements, the agreement analysis showed relatively wide upper limits of agreement (LoA), particularly for radial inclination measurements (≥ 10°, Fig. 4). Although narrower LoA are typically preferred, and our LoA for volar tilt measurements exceeded the previously established threshold of clinically acceptable deviation of up to 5°^14^, methodological uncertainties must be considered when interpreting the results. The threshold of 5° was derived from conventional measurements in 2D radiographs, which showed a standard deviation of 3.9° in repeated measurements by an experienced rater^7^ and LoA range between two raters of 8° for volar tilt and 11° for radial inclination^11^. Furthermore, the impact of projection errors during acquisition can introduce up to 6° of variability in volar tilt measurements^6^. Since our algorithm had 100% reproducibility, we mainly attribute the wide LoA to the variability of the 2D reference, rather than to errors in 3D. Although we aimed to minimise the effect of interobserver variability in the conventional measurements by conducting a consensus reading, we acknowledge that consensus may mask underlying variability between observers. Further studies with a larger sample size, however, are necessary to establish a more certain LoA, to ensure that the proposed method is suitable for use in clinical practice.

Our LoA between manual and automatic 3D measurements of volar tilt (−6.6°, 6.1°, Fig. 5) corresponded to those reported by Winter et al.^20^ (6.2°) but were wider for radial inclination (−3.5°−5.4° compared to 1.8°) for both malunited and healthy radii. LoA between manual 2D and automatic 3D measurements were also generally wider than the LoA between automatic 3D and manual 3D measurements reported by Winter et al. in another study^19^. Significant methodological differences likely explain these discrepancies. In the latter study, Winter et al. defined the Z-axis of the 3D coordinate system using the ulnar diaphysis and measured volar tilt and radial inclination as angles of the entire distal articular surface, making their method fundamentally different from the conventional approach.

Accurate landmark placement to automatically define anatomical CS is critical for accurately estimating radiographic parameters on 3D models. In the current study, the largest mean Euclidean distance between corresponding landmarks placed by any rater and the algorithm was 2.7 mm for the dorsal rim landmark, and the largest mean angle discrepancy in the Z-axis was 2.5°. Our discrepancies were generally larger than the median of up to 1.1 mm for radial landmarks and 0.6° for the Z-axis reported by van Loon et al.^22^ and the mean discrepancies of up to 0.8 mm and 1° between manual raters and the algorithm reported by Velasquez Garcia et al.^23^. Both these studies, however, were conducted on healthy radius models. Our results were comparable to those reported by Winter et al.^20^, who also included both malunited and healthy radii in their study. Again, the differences between studies may be attributed to the varying designs in landmark placement analysis. Velasquez Garcia et al.^23^, similarly to us, reported differences between each pair of the three raters. Winter et al.^20^ quantified reliability as the radius of the sphere enclosing repeated placements of a landmark by different raters, whereas van Loon et al.^22^ used the mean position of manually placed landmarks as the reference. In another study, de Roo et al.^21^ assessed coordinate system displacement rather than landmark displacement, using the automatically determined coordinate system as the reference.

An essential aspect of measuring landmark discrepancy is considering both its magnitude and direction^22^. A positive relationship between landmark discrepancy and differences in radiographic parameters might be expected. However, we did not observe such a relationship, as only the magnitude of discrepancy was considered in our analysis. Determining the direction of landmark displacement would require the definition of a true CS. Although the algorithm has the potential to serve as a reliable reference for the anatomical CS in radius models, this assumption was not applied at the validation stage. Moreover, because manual landmark placement and CS definition are subject to inter-rater variability^20–22^ (Table 3), the direction of landmark displacement was not analysed in this study.

The main limitation of our study is the limited generalizability of the results, due to the small sample size and the limited number of raters. Additionally, the analysis was limited to radii with extra-articular malunions. Although we expect the algorithm to work well for any radius model that is one entity, further research is necessary to validate the algorithm in other cohorts, such as patients with acute fractures. Our future study will also assess the impact of CT image resolution on the accuracy of the automatic measurements. However, by releasing the algorithm publicly, we encourage independent validation. The radiographic measurements on 3D models are also currently limited to only volar tilt and radial inclination. In the future, we plan to expand the algorithm to include measurements of ulnar variance and axial rotation. Further studies should also investigate whether 3D measurements offer improved prediction of clinical outcomes compared to conventional 2D measurements^37^.

## Conclusions

Transitioning from 2D images to 3D models provides a precise method for evaluating distal radius deformity. Our algorithmic approach eliminates the limitations imposed by inter-rater variability in manual 3D measurements. By developing an automatic 3D method aligned with established 2D CS and landmarks, this study overcomes key limitations of previous approaches. The algorithm demonstrated high reproducibility and close agreement with 2D measurements, supporting its potential for future clinical use.

## Supplementary Information

Below is the link to the electronic supplementary material.

Supplementary Material 1

## Data Availability

The data used in this article are available from the corresponding author on reasonable request.

## References

[1] de Muinck Keizer, R. J. O. et al. Three-dimensional virtual planning of corrective osteotomies of distal radius malunions: a systematic review and meta-analysis. *Strat Traum Limb Recon*. **12**, 77–89 (2017). 10.1007/s11751-017-0284-8 PMC5505881 28444580

[2] Meesters, A. M. L., Assink, N. & IJpma, F. F. A. Functional outcome of 2-D- and 3-D-guided corrective forearm osteotomies: a systematic review. *J. Hand Surg. Eur. Vol*. **49**, 843–851 (2024). 37747738 10.1177/17531934231201962 PMC11264531

[3] Buijze, G. A., Verstreken, A. & Verstreken, F. Role of Three-Dimensional guides in management of forearm and wrist malunions. *Hand Clin.* **40**, 89–95 (2024). 37979993 10.1016/j.hcl.2023.09.002

[4] Bodansky, D. M. S. et al. Insights and trends review: the role of three-dimensional technology in upper extremity surgery. *J. Hand Surg. Eur. Vol*. **48**, 383–395 (2023). 36748271 10.1177/17531934221150498

[5] Buijze, G. A. et al. Three-Dimensional compared with Two-Dimensional preoperative planning of corrective osteotomy for Extra-Articular distal radial malunion: A multicenter randomized controlled trial. *J. Bone Joint Surg. Am.* **100**, 1191–1202 (2018). 30020124 10.2106/JBJS.17.00544

[6] Suojärvi, N. & Waris, E. Radiographic measurements in distal radius fracture evaluation: a review of current techniques and a recommendation for standardization. *Acta Radiol.* **65**, 1065–1079 (2024). 39043232 10.1177/02841851241266369

[7] Fox, S., Johnston, G. & Stewart, S. Improved precision of radiographic measurements for distal radius fractures after a technique-teaching tutorial. *Can. J. Surg.* **63**, E261–E271 (2020). 32436686 10.1503/cjs.001419 PMC7828995

[8] THOMASON, K. & SMITH, K. L. The reliability of measurements taken from Computer-Stored digitalised X-Rays of acute distal radius fractures. *J. Hand Surg. Eur. Vol*. **33**, 369–372 (2008). 18562374 10.1177/1753193407087509

[9] Kreder, H. J. et al. X-ray film measurements for healed distal radius fractures. *J. Hand Surg. Am.* **21**, 31–39 (1996). 8775193 10.1016/S0363-5023(96)80151-1

[10] Macdermid, J. C. et al. Reliability of hand fellows’ measurements and classifications from radiographs of distal radius fractures. *Can. J. Plast. Surg.* **9**, 51–58 (2001).

[11] Jensen, J. et al. Dorsal Tilt of the distal radius fracture changes with forearm rotation when measured on radiographs. *J. Hand Surg. Glob Online*. **3**, 182–189 (2021). 35415563 10.1016/j.jhsg.2021.05.006 PMC8991546

[12] Zanetti, M., Gilula, L. A., Jacob, H. A. C. & Hodler, J. Palmar Tilt of the distal radius: influence of Off-lateral Projection— initial observations. *Radiology* **220**, 594–600 (2001). 11526254 10.1148/radiol.2202001699

[13] Cirpar, M., Gudemez, E., Cetik, O., Turker, M. & Eksioglu, F. Rotational deformity affects radiographic measurements in distal radius malunion. *Eur. J. Orthop. Surg. Traumatol.* **21**, 13–20 (2011).

[14] Prommersberger, K. J., Van Schoonhoven, J. & Lanz, U. B. Outcome after corrective osteotomy for malunited fractures of the distal end of the radius. *J. Hand Surg. Br.* **27**, 55–60 (2002). 11895348 10.1054/jhsb.2001.0693

[15] McQueen, M. & Caspers, J. Colles fracture: does the anatomical result affect the final function? *J. Bone Joint Surg. Br.* **70**, 649–651 (1988). 3403617 10.1302/0301-620X.70B4.3403617

[16] Aro, H. T. & Koivunen, T. Minor axial shortening of the radius affects outcome of colles’ fracture treatment. *J. Hand Surg. Am.* **16**, 392–398 (1991). 1861016 10.1016/0363-5023(91)90003-t

[17] Smees, C. J. et al. A comparison of 3-D CT and 2-D plain radiograph measurements of the wrist in extra-articular malunited fractures of the distal radius. *J. Hand Surg. Eur. Vol*. **49**, 546–553 (2024). 37987680 10.1177/17531934231213790 PMC11044515

[18] Miyake, J. et al. Comparison of three dimensional and radiographic measurements in the analysis of distal radius malunion. *J. Hand Surg. Eur. Vol*. **38**, 133–143 (2013). 22736743 10.1177/1753193412451383

[19] Winter, R. et al. Validation of an automatic three-dimensional method for distal radius measurements and malunion quantification. *J. Hand Surg. Eur. Vol*10.1177/17531934251340240 (2025). 40376982 10.1177/17531934251340240

[20] Winter, R. et al. An Evaluation of the Reliability of Manual Landmark Identification on 3D Segmented Wrists. *The J. Bone Joint Surgery* **106**, 315–322 (2024). 10.2106/JBJS.23.00173 37995208

[21] de Roo, M. G. A. et al. Accuracy of manual and automatic placement of an anatomical coordinate system for the full or partial radius in 3D space. *Sci. Rep.* **10**, 8114 (2020). 32415290 10.1038/s41598-020-65060-7 PMC7229017

[22] van Loon, D. F. R., van Es, E. M., Eygendaal, D., Veeger, D. H. E. J. & Colaris, J. W. Automatic identification of radius and ulna bone landmarks on 3D virtual models. *Comput. Biol. Med.* **179**, 108891 (2024). 39047505 10.1016/j.compbiomed.2024.108891

[23] Velasquez Garcia, A. et al. Automated coordinate system estimation: A preliminary step toward computer-assisted radial head arthroplasty planning. *J. Orthop. Res.* **43**, 348–361 (2025). 39446984 10.1002/jor.25996

[24] Suojärvi, N., Tampio, J., Lindfors, N. & Waris, E. Computer-aided 3D analysis of anatomy and radiographic parameters of the distal radius. *Clin. Anat.* **34**, 574–580 (2020). 32346905 10.1002/ca.23615

[25] Wu, G. et al. ISB recommendation on definitions of joint coordinate systems of various joints for the reporting of human joint motion—Part II: shoulder, elbow, wrist and hand. *J. Biomech.* **38**, 981–992 (2005). 15844264 10.1016/j.jbiomech.2004.05.042

[26] CT Scan Protocol -. Osteotomies - Upper Extremity - English - L-102000-01.pdf.

[27] Tang, J. B. & Giddins, G. Why and how to report surgeons’ levels of expertise. *J. Hand Surg. Eur. Vol*. **41**, 365–366 (2016). 27076602 10.1177/1753193416641590

[28] Medoff, R. J. Essential radiographic evaluation for distal radius fractures. *Hand Clin.* **21**, 279–288 (2005). 16039439 10.1016/j.hcl.2005.02.008

[29] Suojärvi, N., Lindfors, N., Höglund, T., Sippo, R. & Waris, E. Radiographic measurements of the normal distal radius: reliability of computer-aided CT versus physicians’ radiograph interpretation. *J. Hand Surg. Eur. Vol*. **46**, 176–183 (2021). 33148107 10.1177/1753193420968399

[30] Martin Bland, J. & Altman, D. G. Statistical methods for assessing agreement between two methods of clinical measurement. *Lancet* **327**, 307–310 (1986). 2868172

[31] D’Agostino, R. B. An omnibus test of normality for moderate and large size samples. *Biometrika* **58**, 341–348 (1971).

[32] D’Agostino, R. & Pearson, E. S. Tests for departure from Normality. Empirical results for the distributions of b2 and √ b1. *Biometrika* **60**, 613–622 (1973).

[33] Bland, J. M. & Altman, D. G. Measuring agreement in method comparison studies. *Stat. Methods Med. Res.* **8**, 135–160 (1999). 10501650 10.1177/096228029900800204

[34] Sample size for. a study of agreement between two methods of measurement. https://www-users.york.ac.uk/~mb55/meas/sizemeth.htm.

[35] Athlani, L., Chenel, Berton, A., Detammaecker, R. & Dautel, G. Three-Dimensional versus radiographic measurements for analyzing Extra-Articular distal radius malunion. *J. Hand. Surg.* **45**, 984e1–984e7 (2020). 10.1016/j.jhsa.2020.03.009 32327340

[36] Schindele, S., Oyewale, M., Marks, M., Brodbeck, M. & Herren, B. D Three-Dimensionally planned and printed Patient-Tailored plates for corrective osteotomies of the distal radius and forearm. *J. Hand. Surg.* **49**, 277e1–277e8 (2024). 10.1016/j.jhsa.2022.06.021 35985863

[37] Plant, C. E., Parsons, N. R. & Costa, M. L. Do radiological and functional outcomes correlate for fractures of the distal radius? *Bone Joint J.* **99-B**, 376–382 (2017). 28249979 10.1302/0301-620X.99B3.35819

