# Supplementary material for: Automatic measurements of volar tilt and radial inclination of the distal radius on 3D models: validation against manual methods
Source: Sci Rep. 2026 Jan 6;16:810. doi: 10.1038/s41598-025-33737-6 (PMC12779976; doi:10.1038/s41598-025-33737-6)
Supplement: Supplementary file 1 — Supplementary Material 1 [file 41598_2025_33737_MOESM1_ESM.pdf]

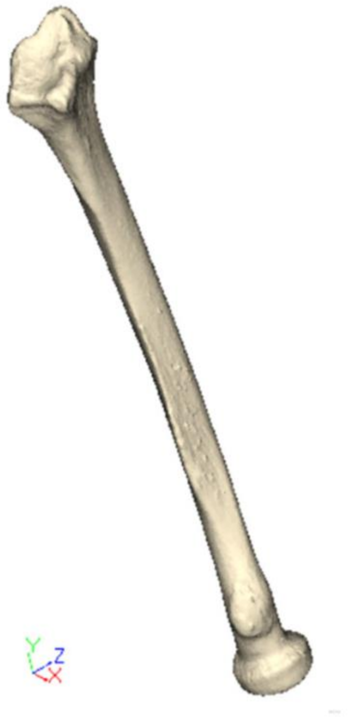	The model's initial position when imported into the program.
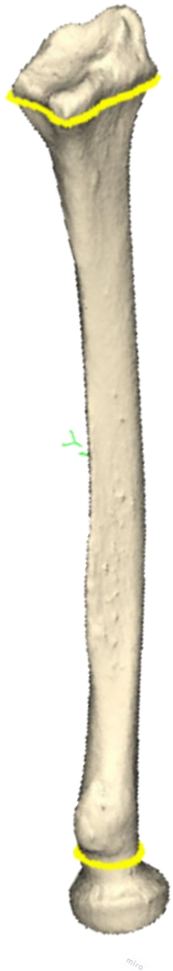	Step 2. Identifying the distal and proximal ends of the radius.

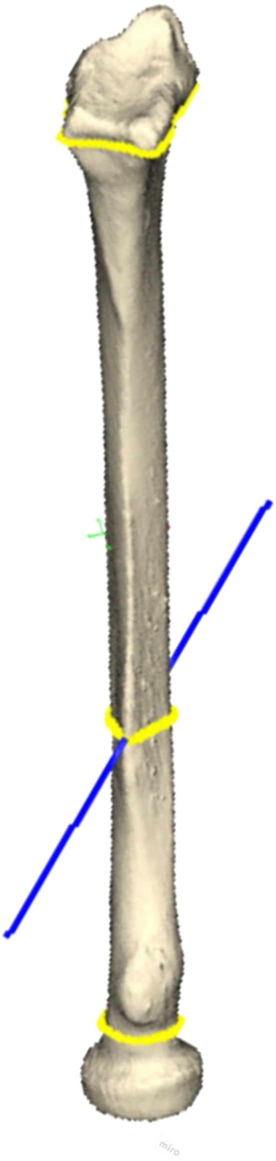	Step 3. Identifying the radial and ulnar directions.

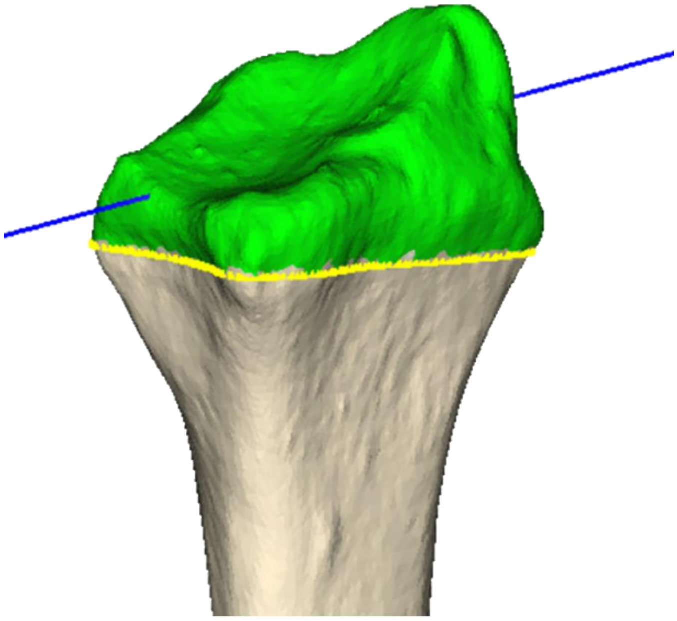	Step 4. Identifying the distal articular surface and refining the X-axis in the radio-ulnar direction.
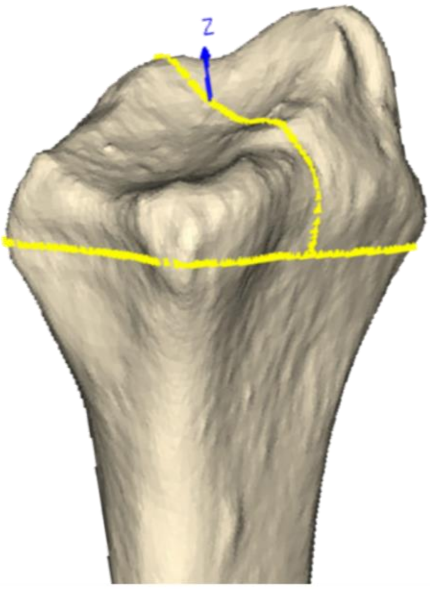	Steps 5-7. Refining the Y-axis in the dorsal-volar direction.

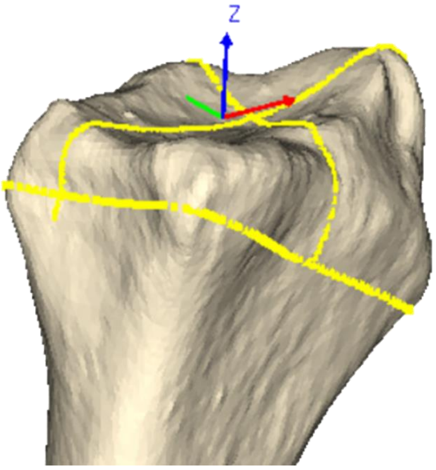	Step 8-9. Identifying the styloid.
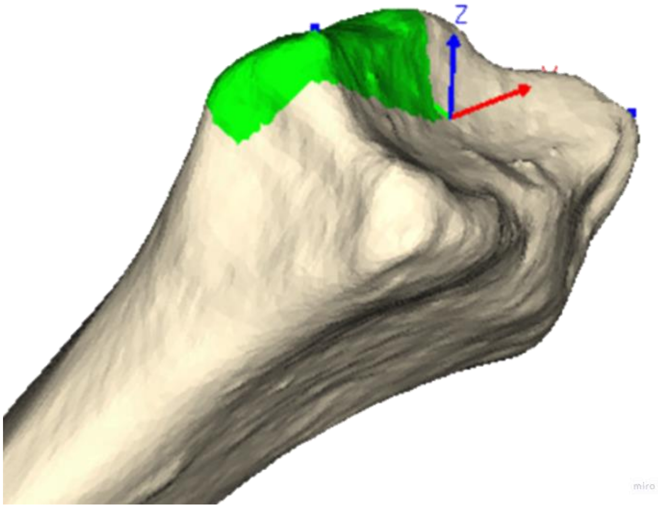	Step 10. Identifying the ulnar dorsal corner.

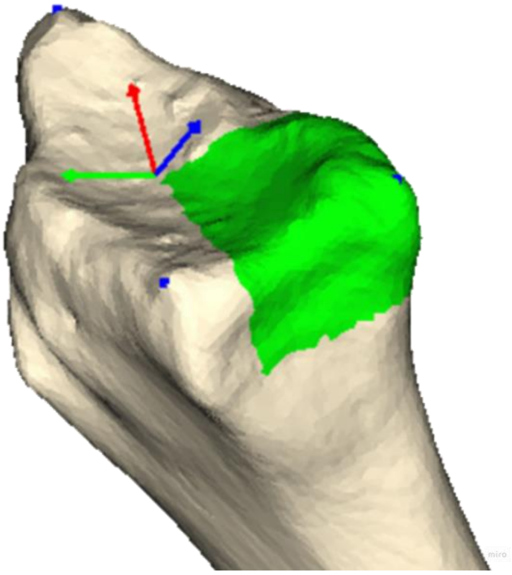	Step 10. Identifying the ulnar volar corner.
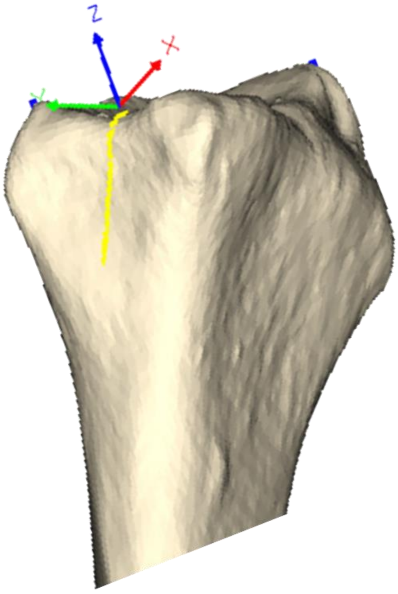	Step 11. Identifying the central reference point.

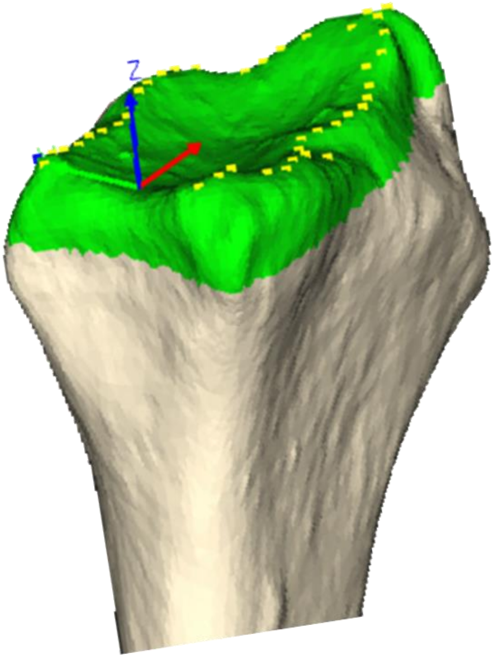	Step 12-13. Identifying the reference points for volar tilt calculation.
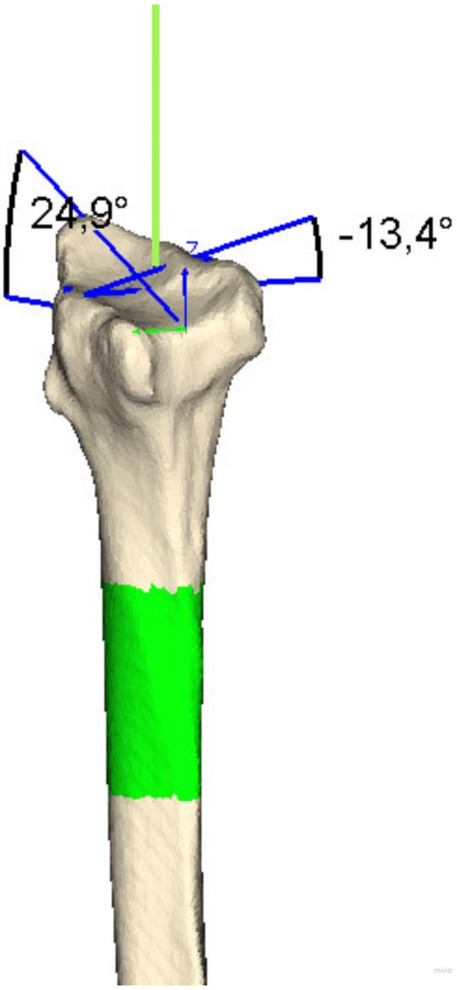	Steps 14-16. Refining the Z-axis direction (green line) and styloid position. Volar tilt and radial inclination are calculated.
